# Evidence that spontaneous reactivation of herpes virus does not occur in mice

**DOI:** 10.1186/1743-422X-2-67

**Published:** 2005-08-18

**Authors:** Bryan M Gebhardt, William P Halford

**Affiliations:** 1 LSU Eye Center, Louisiana State University Health Sciences Center, New Orleans, LA 70112 USA; 2 Department of Veterinary Microbiology, Montana State University, Bozeman, MT 59718 USA

## Abstract

**Background:**

Some species, including humans and rabbits, exhibit periodic viral reactivation and shed infectious virus at the infected end organ. Mice may be an exception, because spontaneous shedding of infectious virus rarely, if ever, occurs. However, spontaneous molecular reactivation, *i.e.*, the expression of a few viral genes and the synthesis of the viral glycoproteins coded for by these genes, has been reported. This finding has prompted the assumption that molecular reactivation is an indicator of reactivation and the production of infectious virus. The goal of this study was to differentiate between viral gene expression during latency and the episodic production of infectious virus in mice.

**Results:**

Viral reactivation and infection were not seen in herpes simplex virus type 1 (HSV-1) latent ganglion graft recipient BALB/c scid or immunocompetent BALB/c mice, which survived the 65-day observation period with no evidence of viral infection although the immunocompetent mice developed cellular and humoral immunity to HSV-1. In contrast, BALB/c scid recipients of ganglia containing reactivating virus invariably developed a local and, subsequently, systemic viral infection and died within 14 days. Immunocompetent BALB/c mice that received ganglion grafts containing reactivating virus survived the infection and became immune to the virus. Trigeminal ganglia removed from scid and immunocompetent recipient graft sites 5, 14, and 28 days after transplantation contained latent virus and viable neurons.

**Conclusion:**

The results suggest that, within the limits of detection of the experiments, spontaneous episodic production of immunogenic viral antigens but not of infectious virus occurs in mouse neural ganglia during latency.

## Background

The infectious cycle of herpes simplex virus type 1 (HSV-1) in experimental animals is similar to that which occurs in humans, but there may be a significant difference as well. HSV-1 readily infects epithelial surfaces of most mammalian species, replicates in these cells, enters the nervous system, and achieves a latent state in neurons in the peripheral nervous system. A notable species difference is that the virus undergoes spontaneous, episodic reactivation with or without evidence of recurrent disease in humans and rabbits, whereas mice either do not undergo spontaneous reactivation or undergo spontaneous reactivation at such a low frequency that it is difficult to document [1].

Testing an end organ such as the eye or the site of viral latency, the sensory ganglia, for infectious virus during latency in mice fails to yield virus [2-5]. However, evidence of viral gene expression in the trigeminal ganglia of mice during latency has been reported [6,7]. In addition to the expression of the latency-associated transcript (LAT), the expression of other viral genes and their products has been found in a small number of ganglion cells. Feldman *et al. *[8] described "abundant" expression of viral genes and proteins and noted viral DNA synthesis in occasional neurons. This process was termed "*spontaneous molecular reactivation*"; no evidence of infectious virus was reported in this study [8].

Stevens and Cook [3] transplanted ganglia from latent mice into mice that were actively immunized with irradiated virus or passively immunized with anti-HSV antibody and concluded that antiviral antibody helped maintain viral latency. Tenser *et al. *[4] reported that viral reactivation occurred in ganglion transplants after *ex vivo *explantation. The occurrence of secondary latency was proposed as a consequence of viral reactivation and infection of "secondary" neurons in the grafts; however, infectious virus was not found in ganglion homogenates [4].

The current study was designed to differentiate between viral gene expression and the production of infectious virus in latent mouse ganglia *in vivo*. The experimental system was designed to assess for the production of small numbers of infectious viral particles which would lead to morbidity and, ultimately, mortality in the host mice. In the results reported here, molecular reactivation (*i.e.*, expression of HSV-1 genes and production of glycoproteins during latency) did not proceed to the production of detectable infectious virus in immune-deficient mice. The results suggest that viral reactivation does not occur spontaneously and episodically in the mouse trigeminal ganglion *in vivo*.

## Results

### Absence of infectious virus in the trigeminal ganglion during latency

Infectious virus was present on the ocular surface and in both trigeminal ganglia of a group of five BALB/c mice sacrificed 5 days after topical ocular infection (Table 1). On days 10, 20, 30, 50, 70, and 100 after infection, both the ocular surface and the trigeminal ganglion homogenates of latently infected mice failed to yield infectious virus as evidenced by cytopathic effect on Vero cells (Table 1).

**Table 1:** Analysis of infectious virus in the eye and trigeminal ganglion during establishment of latency

**Location**	**Days after infection**^**a**^						
	
	**5**	**10**	**20**	**30**	**50**	**70**	**100**
Eye	5/5^b^	0/5	0/5	0/5	0/5	0/5	0/5
Trigeminal ganglia	10/10	0/10	0/10	0/10	0/10	0/10	0/10

^a^Infected mice (N = 5) were killed and eye swabs (both combined) and trigeminal ganglion homogenates (separately) tested for infectious virus.

^b^The numbers indicate number of eye swabs and trigeminal ganglion homogenates containing infectious virus/number of each tested at each time.

### Sensitivity of the ganglion assay

Assay of trigeminal ganglion homogenates for infectious virus immediately after microinjection of a known number of PFU of virus revealed that the limit of sensitivity for the *in vitro *assay was between 50 and 100 PFU per ganglion (Table 2). All ganglia injected with 100 PFU yielded plaques and 6 of 10 ganglia injected with 50 PFU yielded plaques (Table 2). Injection of smaller numbers of PFU did not reproducibly yield plaques (Table 2).

**Table 2:** Detection of virus injected into the trigeminal ganglion by plaque assay^a^

**Number of PFU injected/ganglion**	**Number of ganglia containing infectious virus/number of ganglia tested**	**Mean PFU ± standard deviation**
100	10/10	28 ± 7
50	6/10	7 ± 4
10	1/10	1
5	0/10	0
1	0/10	0

^a^Intact trigeminal ganglia were injected with the amounts of virus indicated and homogenized. The homogenate was tested for infectious virus by plaque assay.

Ten out of 10 of the BALB/c scid mice receiving ganglia injected with 100 PFU and 9 of 10 mice receiving ganglia injected with 50 PFU died from complications of viral pathogenesis within 12 days of receiving ganglion transplants (Table 3). Five of 10 BALB/c scid mice receiving ganglion grafts containing 10 PFU and 1 of 10 mice receiving grafts containing 5 PFU died within 14 days of receiving the grafts (Table 3). None of the 10 animals receiving the ganglion grafts containing 1 PFU gave evidence of viral infection and viral pathogenesis over a 35-day observation period.

**Table 3:** Detection of virus injected into the trigeminal ganglion by transplantation into scid mice^a^

**Number of PFU injected/ganglion**	**Number of mice dead/number of mice grafted**
100	10/10
50	9/10
10	5/10
5	1/10
1	0/10

^a^Trigeminal ganglia injected with the amount of virus indicated were transplanted to BALB/c scid mice and the animals observed for viral pathogenesis and death over a 35-day period.

### Outcome of ganglion transplantation

#### Acute protocol

**Figure 1:**
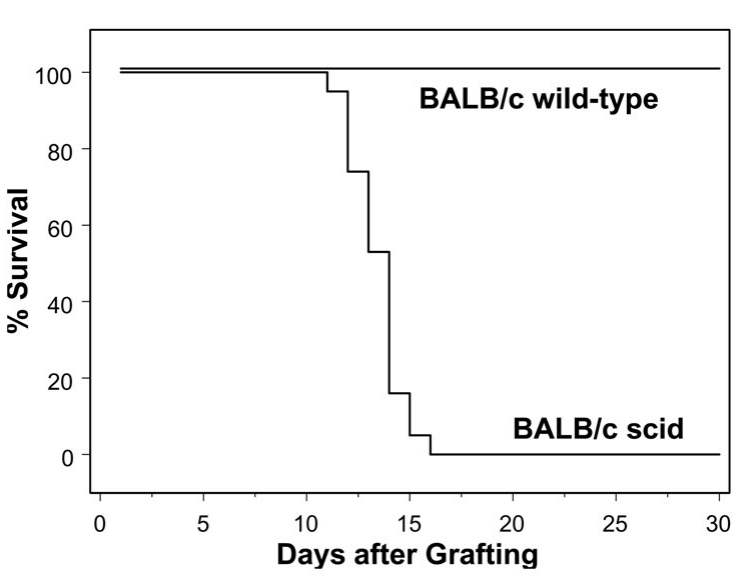
**Acute Protocol: Kaplan Meier analysis of the fate of ganglion transplant recipients. **BALB/c scid mice (N = 19) and immunocompetent BALB/c mice (N = 15) were observed daily for evidence of infection and death. The day of death was recorded as the number of days after grafting. BALB/c scid recipients all died by day 16, whereas all of the immunocompetent BALB/c recipients survived throughout the entire observation period (*P *< 0.0001).

Results from three replicate experiments revealed that the BALB/c scid recipients of ganglion transplants from acutely infected BALB/c donors transplanted 3 days after infection (N = 19) all died, with a mean time to death of 14 days and a standard deviation of ± 2 days (Fig. 1). In contrast, all of the immunocompetent BALB/c recipients of acutely infected ganglia (N = 15) survived (Fig. 1).

The cause of death in the BALB/c scid mice was not extensively examined in this study. As the animals became progressively moribund, it was evident that they were experiencing a neurological disease resembling encephalitis. In randomly chosen animals, virus was isolated from the ear graft site at the time that the animal died. Confirmation that virus was replicating at the transplant site is provided below.

#### Latent protocol

**Figure 2:**
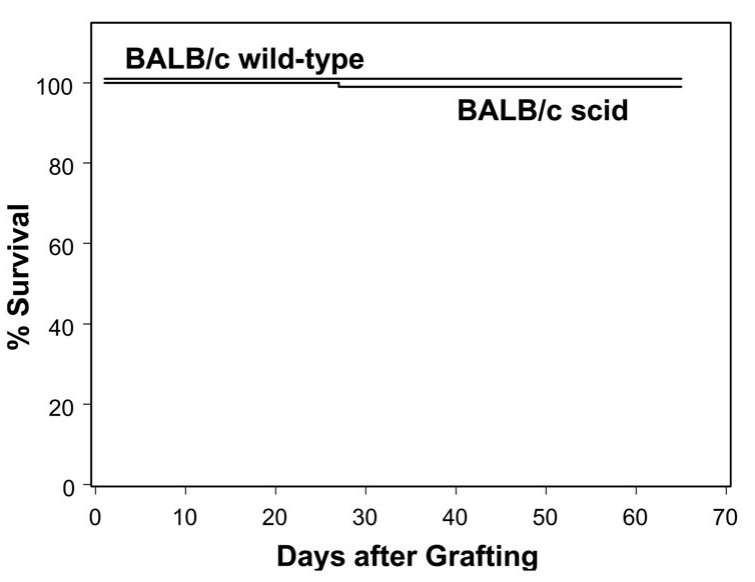
**Latent Protocol: Kaplan Meier analysis of the fate of BALB/c scid (N = 70) and immunocompetent BALB/c (N = 30) ganglion recipients. **Mice were observed daily for evidence of infection and death. The day of death was recorded as the number of days after grafting. One of the BALB/c scid mice died on day28, but virus was not found in the graft site or brain of this animal. There was no significant difference between the survival of the BALB/c scid and immunocompetent BALB/c mice (*P *> 0.05).

In a series of three experiments involving a total of 100 recipients (70 BALB/c scid, 30 immunocompetent BALB/c), only 1 of the 70 BALB/c scid recipients of a latent ganglion transplant died (28 days after grafting). Virus was not isolated from the graft site or the brain of this animal. As shown in Figure 2, the remainder of the BALB/c scid recipients survived without evidence of viral infection up to 65 days after grafting. All of the 30 immunocompetent BALB/c recipients of latent ganglion transplants survived for the duration of the experiment (Fig. 2). Mice in both of these groups were bled at 21, 45, and 65 days after grafting to test their sera for anti-HSV-1 antibodies, as described below.

#### Reactivation protocol

**Figure 3:**
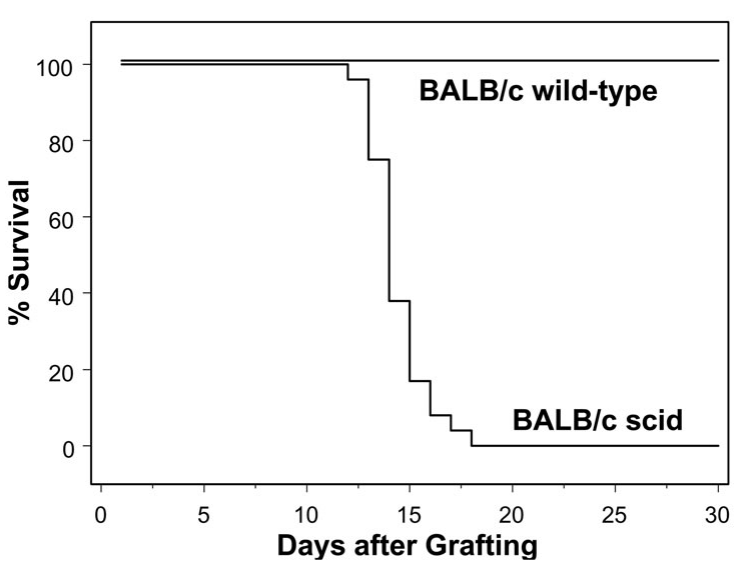
**Reactivation Protocol: Kaplan Meier analysis of the fate of BALB/c scid (N = 24) and immunocompetent BALB/c (N = 16) recipients of ganglion grafts. **Mice were observed daily for infection and death. The day of death was recorded as the number of days after grafting. All of the BALB/c scid recipients were dead by day 18, whereas all of the immunocompetent BALB/c recipients survived throughout the entire observation period (*P *< 0.0001).

Ganglia from BALB/c mice latent for HSV-1 were passaged in tissue culture for 3 days and then transplanted into BALB/c scid recipients (N = 24) and immunocompetent BALB/c recipients (N = 16). All of the BALB/c scid recipients developed viral infections and viral-mediated neurologic disease and died, with a mean time to death of 14 days (Fig. 3). Analyses of the transplanted tissue and the local graft site supported the conclusion that the cause of death was the reactivated virus. In contrast, all of the immunocompetent BALB/c recipients of reactivated ganglia survived without evidence of disease (Fig. 3) and ultimately became immune to the virus.

### Serum anti-HSV-1 antibody responses in ganglion recipients

**Figure 4:**
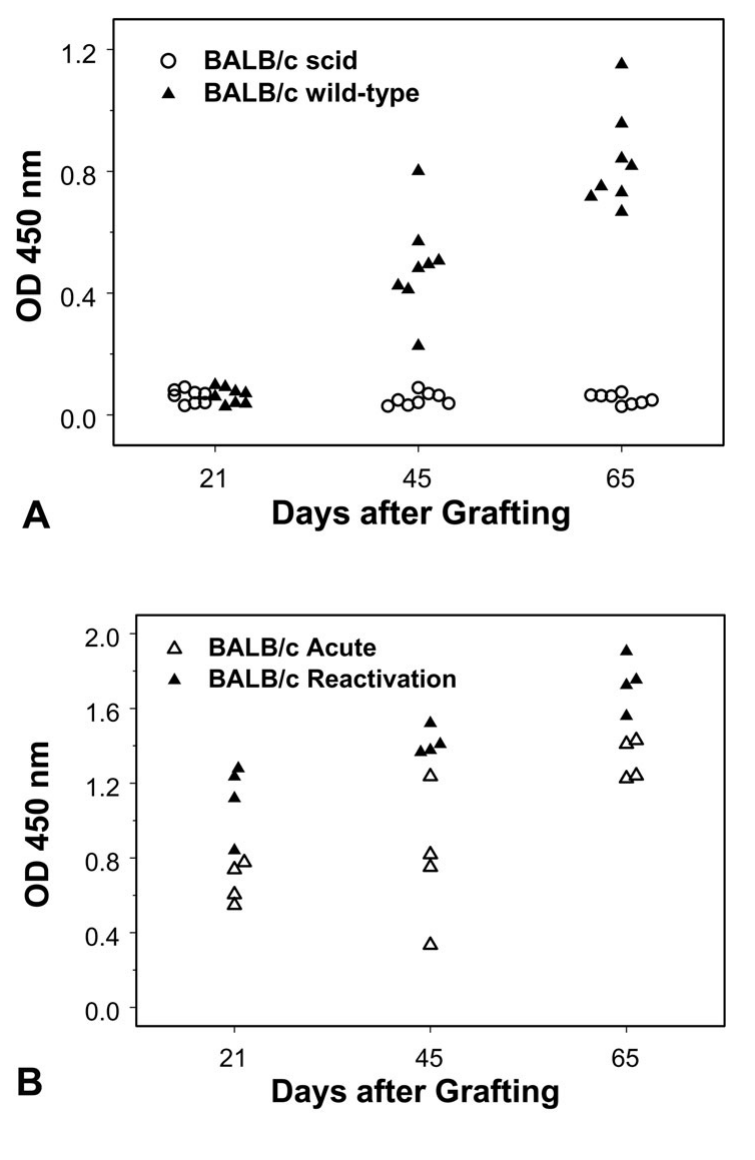
**Serum anti-HSV-1 antibody responses. (a) **Serum anti-HSV-1 antibody responses of BALB/c scid (N = 8) and immunocompetent BALB/c (N = 8) mice in the Latent Protocol. The mice were grafted with ganglia containing latent virus and randomly chosen mice were bled on days 21, 45, and 65 after transplantation. The corrected optical density readings indicate that the BALB/c scid mice did not produce IgG antibody, whereas the immunocompetent BALB/c mice all had serum IgG anti-HSV-1 antibody on days 45 and 65 after transplantation. **(b) **Serum antibody responses of BALB/c mice receiving acutely infected ganglia (N = 4) or ganglia containing reactivating virus (N = 4). The optical density readings indicate that the immunocompetent mice in the Acute Protocol and the Reactivation Protocol developed serum anti-HSV-1 antibody titers by day 21 after grafting and that this antibody continued to be present on days 45 and 65 after transplantation. At each of the three time points, symbols representing one mouse each are spread out to avoid concealment by overlap.

None of the BALB/c scid recipients of latently infected ganglia developed a serum IgG antibody response as measured by enzyme-linked immunosorbent assay (ELISA). Randomly chosen BALB/c scid mice tested at 21, 45, and 65 days after transplantation showed no evidence of having developed a humoral immune response to the virus (Fig. 4a). The BALB/c mice that received latent ganglion transplants did not exhibit an anti-HSV-1 antibody response on day 21 after transplantation, but had serum antibody on days 45 and 65 after grafting (Fig. 4a). The immunocompetent BALB/c recipients of acutely infected ganglia or of ganglia containing reactivating virus developed serum antibody IgG responses by 21 days after infection, which were present also at 45 and 65 days after transplantation (Fig. 4b).

### Cell-mediated immunity in ganglion transplant recipients

**Figure 5:**
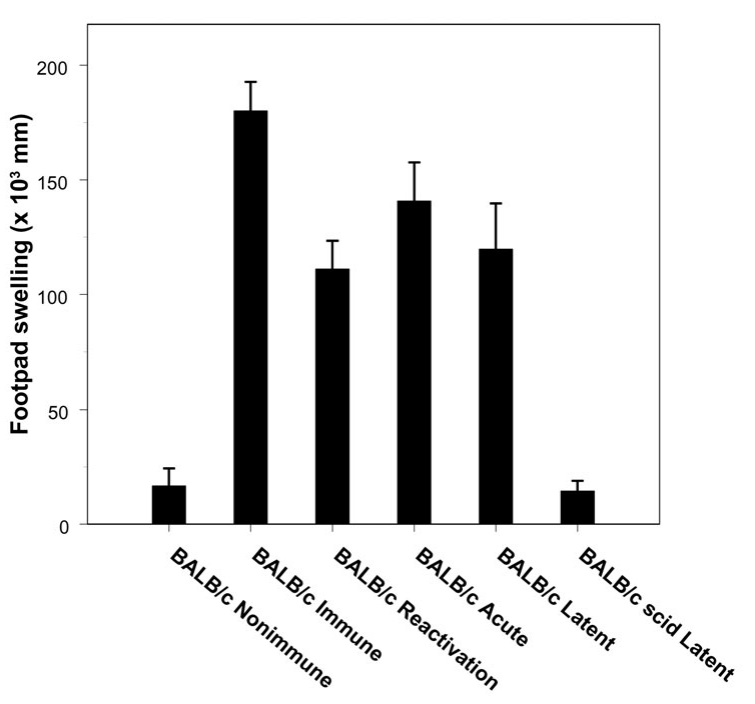
**Footpad swelling responses to measure cell-mediated immunity. **Footpad swelling responses in the BALB/c scid mice in the Latent Protocol (N = 9) and immunocompetent BALB/c recipients in the Latent (N = 7), Acute (N = 7), and Reactivation (N = 6) Protocols are shown. Included in the data sets are the footpad swelling responses of immune(N = 6) and nonimmune (N = 6) immunocompetent BALB/c mice. The BALB/c scid recipients of latent ganglia failed to develop a cell-mediated immune response, whereas four of the seven BALB/c wild-type recipients of latent ganglia showed a delayed-type hypersensitivity response. All of the immunocompetent BALB/c recipients of ganglia in the Acute and Reactivation Protocols exhibited cellular immune reactivity. Comparison of the footpad swelling response of the BALB/c immune mice, immunocompetent BALB/c recipients in the Reactivation Protocol and the Acute Protocol, and immunocompetent BALB/c recipients in the Latent Protocol with the BALB/c scid recipients in the Latent Protocol revealed that the response in the immunocompetent mice in each group was significantly greater than the response in the BALB/c scid mice (*P *< 0.001). Values are means ± SD.

BALB/c scid mice from the Latent Protocol that survived the 65-day observation period were tested by footpad swelling assay. None of the animals tested gave evidence of a delayed-type hypersensitivity response to viral antigens (Fig. 5). In contrast, all of the immunocompetent BALB/c recipients of acutely infected ganglia and recipients of ganglia undergoing viral reactivation exhibited delayed-type hypersensitivity responses on day 65 after ganglion transplantation (Fig. 5). Four of seven immunocompetent BALB/c recipients of latent ganglia also had positive delayed-type hypersensitivity responses on day 65 after transplantation (Fig. 5).

### Viability of the ganglion transplants

Latent ganglia that had been transplanted into BALB/c scid and immunocompetent BALB/c recipients were removed from the graft recipients on days 5, 14, or 28 after transplantation and placed into tissue culture. Seventeen of 18 latent ganglia removed from BALB/c scid animals underwent reactivation *in vitro *(Table 4), typically within 3 to 6 days after explantation. All of the latent ganglia recovered from immunocompetent BALB/c recipients underwent reactivation in tissue culture between days 6 and 10 after explantation.

**Table 4:** Recovery (reactivation) of virus in explanted ganglion grafts^a^

Source of explants	**Day of explantation relative to day of grafting**		
	
	**5**	**14**	**28**
BALB/c scid Latent Protocol	5/5^b^	6/6	6/7
Immunocompetent BALB/c Latent Protocol	4/4	7/7	5/5

^a^Ganglion grafts were placed into culture on the day indicated and the culture medium tested for infectious virus on days 1, 3, 5, 7, 10, 14, and 21 of incubation.

^b^Number of ganglia from which virus reactivated/number of ganglia tested.

**Figure 6:**
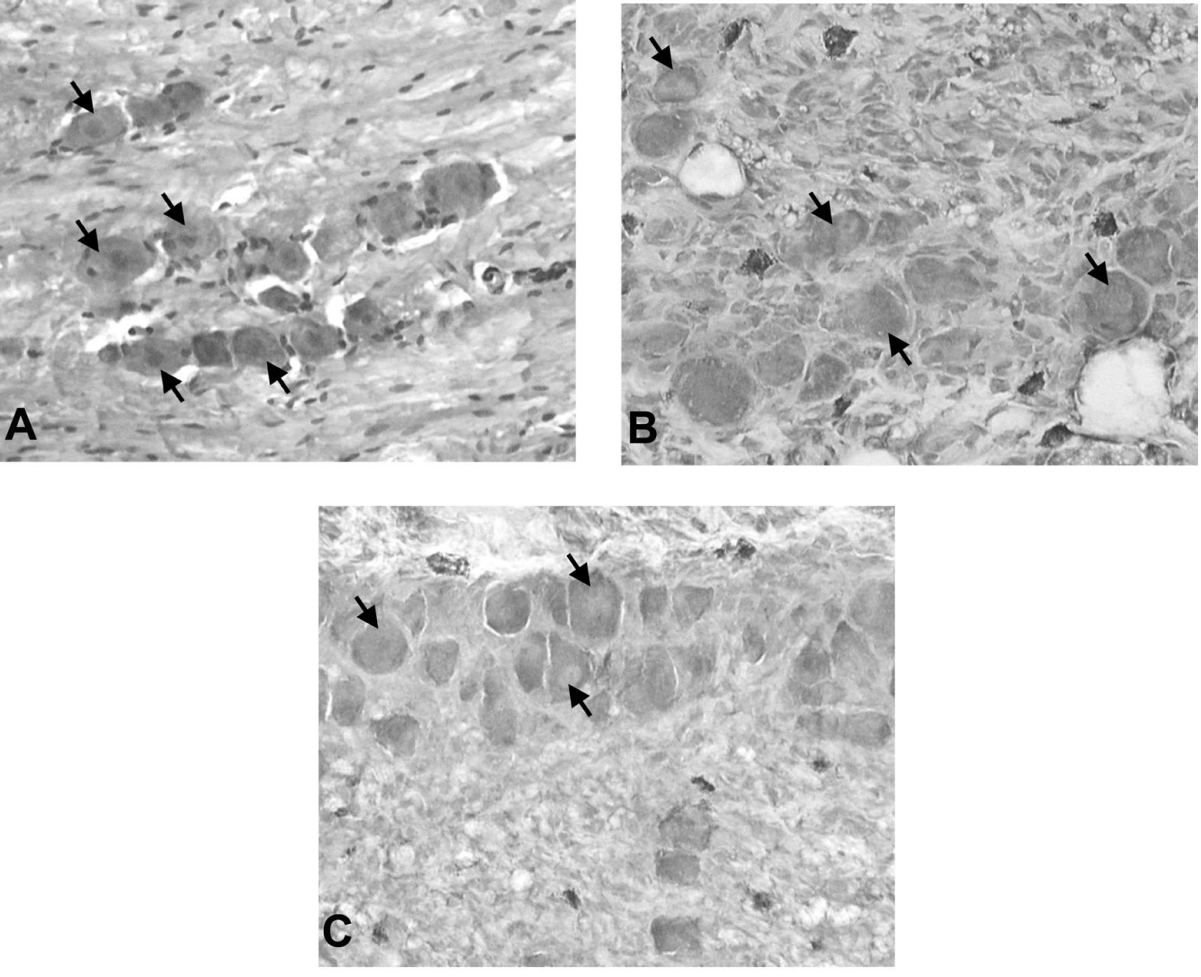
**Histology of ganglion grafts. (a) **Hematoxylin and eosin (H & E) stained section of a freshly isolated trigeminal ganglion. The large neuron cell bodies (arrows) interspersed among a field of nerve fibers present the typical histologic appearance of the trigeminal ganglion. (original magnification 400×) **(b) **H & E stained section of a latent ganglion graft removed from a BALB/c scid mouse 45 days after grafting. In this section, neuron cell bodies (arrows) with typical morphology can be seen. (original magnification 400×) **(c) **H & E stained section of alatent ganglion graft removed from an immunocompetent BALB/c recipient 45 days after transplantation. Clusters of neuron cell bodies (arrows) with typical morphology can be seen. (original magnification 400×)

The histology of ganglion transplants was examined on days 5, 28, and 65 after transplantation. Although the architecture of transplanted ganglia was somewhat altered compared with that of freshly isolated ganglia (Fig. 6a), numerous large cells with the morphology of neurons were seen in latent ganglia transplanted to BALB/c scid (Fig. 6b) and immunocompetent BALB/c (Fig. 6c) recipients.

Table 5 presents the results of the vital dye staining of cells isolated from ganglion transplants. Viable small (non-neuronal) and large (neurons) cells were found in roughly the same ratios on days 5, 14, 28, and 65 after transplantation. These ratios were similar to the ratios of small and large cells obtained from freshly isolated ganglia (Table 5).

**Table 5:** Viability of cells in ganglion grafts^a^

**Source of ganglia**	**Day of cell viability determination relative to day of grafting**							
	
	**5**		**14**		**28**		**65**	
	
	**Small**	**Large**	**Small**	**Large**	**Small**	**Large**	**Small**	**Large**
BALB/c scid Latent Protocol	328/416^b ^(79)	54/72 (75)	477/519 (92)	38/49 (78)	622/705 (88)	88/97 (91)	219/279 (78)	41/54 (76)
Immuno-competent BALB/c Latent Protocol	789/885 (89)	66/81 (81)	413/500 (83)	75/82 (91)	917/998 (92)	31/48 (65)	261/308 (85)	87/101 (86)
Freshly isolated ganglia	419/524 (80)	88/110 (80)	523/567 (92)	101/123 (82)	816/911 (90)	23/38 (61)	377/408 (92)	99/122 (81)

^a^Ganglion grafts or freshly isolated ganglia were dissociated and stained with vital DNA dye and trypan blue. The total numbers of small cells (10–50 μm) and large cells (larger than 50 μm), as well as the number of live cells in each size category, were determined by microscopic examination.

^b^Number of viable cells of each size/total number of cells of each size counted (%).

**Figure 7:**
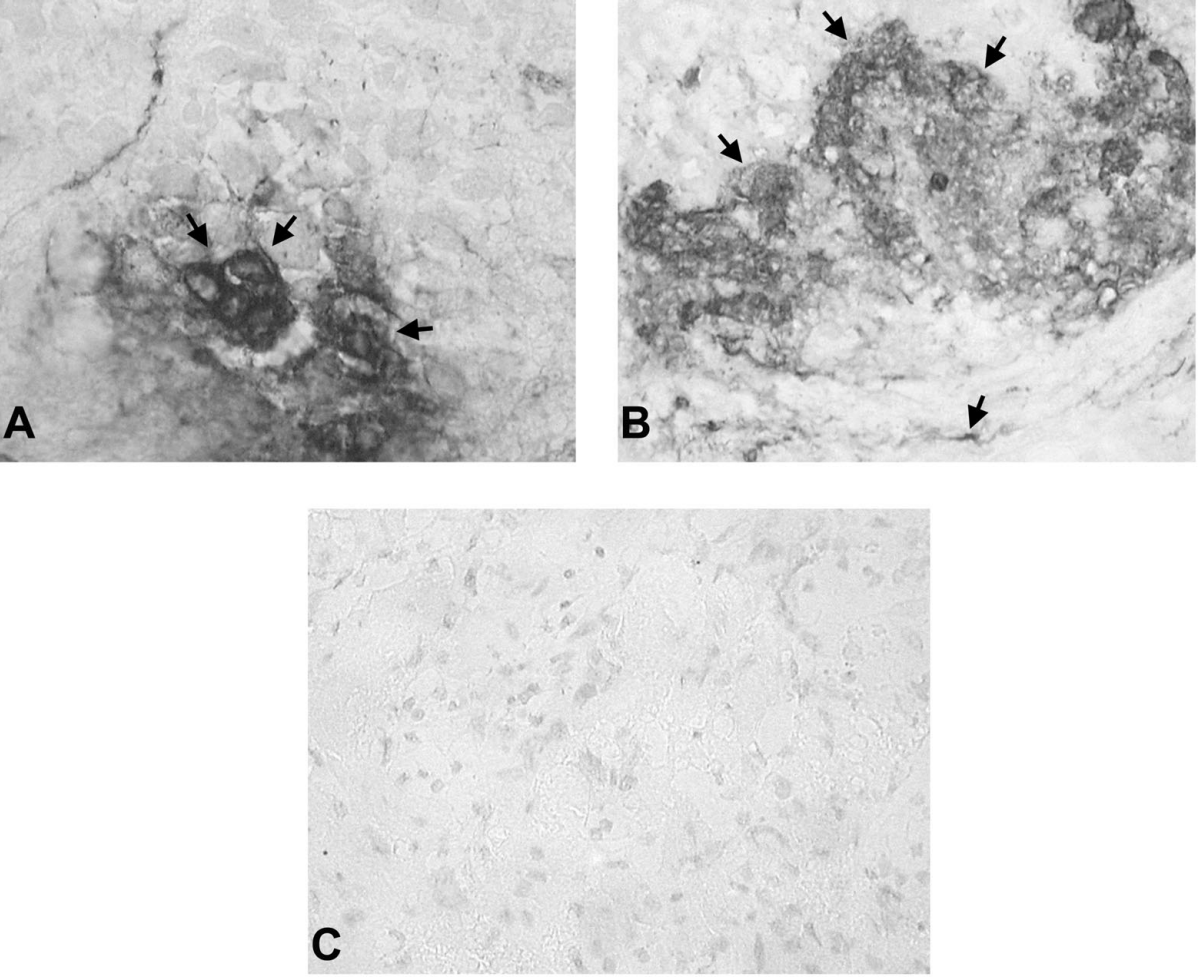
**Immunohistochemical staining for viral antigens. (a) **Immunohistochemical staining of a tissue section through the ear graft site of a BALB/c scid mouse containing a ganglion from an acutely infected donor 7 days after grafting. In this immunoperoxidase-stained section, cells expressing HSV-1 antigens can be seen in the ganglion graft (arrows). (original magnification, 400×) **(b) **Immunohistochemical staining for HSV-1 antigens in the ganglion graft in a BALB/c scid mouse from the Reactivation Protocol. Cells expressing HSV-1 antigen are seen in both the graft and the surrounding ear cells at the graft site (arrows). (original magnification, 400×) **(c) **Immunohistochemical staining of a latent ganglion graft in a BALB/c scid recipient at 28 days after grafting. No evidence of cells expressing HSV-1antigens was seen in these tissue sections. (original magnification, 400×)

Confirmation that viral reactivation and viral glycoprotein synthesis was occurring in ganglia transplanted during acute infection and in ganglia transplanted following reactivation was obtained by performing immunohistochemical staining for HSV-1 antigens in tissue sections. Staining of viral antigens was seen in the ganglion transplants and adjacent ear cells in BALB/c scid recipients of acutely infected (Fig. 7a) and reactivating (Fig. 7b) ganglia, but not in recipients of latent ganglion transplants (Fig. 7c).

## Discussion

The results of these experiments indicate that little, if any, infectious virus is produced during latency in mice. It has been proposed that some component of the immune system is necessary to induce HSV-1 into latency and prevent viral reactivation [9-15]. Sawtell [16] reported that immune cells in mouse ganglia do not inhibit viral reactivation. Thus, the role of antiviral immunity in the establishment and maintenance of latency is still being debated.

The immunocompetent BALB/c mice in the acute, latent, and reactivation protocols developed cellular and humoral immunity, indicating that there was an adequate amount of viral antigen produced in all three circumstances to sensitize the recipients. The finding that T cell-mediated and humoral responses developed and were sustained for 65 days suggests that viral antigen expression during latency has a role in this process. BALB/c scid mice lack an acquired immune system but have an intact innate immune system, including cells such as macrophages and natural killer (NK) cells and antiviral cytokines such as the types 1 and 2 interferons. NK cells alone cannot protect scid mice from HSV-1 infection and pathogenesis [17]. However, protection against viral-mediated death can be provided by T lymphocytes [18-21]. There is ample evidence to indicate that the interferons modulate the level of viral replication and spread, although this response is not known to protect scid mice from HSV-1-mediated death [22-24]. However, in the absence of an acquired immune system and, in particular, T lymphocytes, the virus evades the interferon response, enters the nervous system, and replicates in vital cells causing a fatal encephalitis.

The findings of the current study appear to imply that mouse neural tissue containing latent HSV-1 (*e.g.*, the trigeminal ganglion) does not support periodic episodic viral reactivation. Although spontaneous molecular viral reactivation has been reported [8], the results presented here suggest that this molecular reactivation does not proceed to the production of detectable infectious virus. It may be that there are nonimmunological cellular or molecular factors that prevent spontaneous viral reactivation in mice.

These findings *in vivo *are particularly important since it is known that explanted mouse neural tissues latent for HSV-1 demonstrate viral reactivation in culture. This suggests that explantation itself or factors in tissue culture that we do not understand may suppress or destroy the *in vivo *factors that maintain viral latency.

A number of reports describe the induction of viral reactivation from latency in mice using a variety of stimuli such as immunosuppressive drugs, UV irradiation, and thermal stress [25-29]. These induction protocols yield a variable frequency of viral reactivation. There are no reports confirming spontaneous episodic shedding of virus at epithelial surfaces of mice, including the eye and genitalia, although it has been reported recently that infectious virus is present in the trigeminal ganglion up to 240 days after infection [16].

The possibility that the surgical trauma of ganglion transplantation or the site in which the transplant was placed (the ear) prevents viral reactivation from occurring and/or prevents infectious virus from leaving this site to infect the animal's nervous system must be considered. However, in the Acute and Reactivation Protocol mice, it was found that ganglia containing infectious virus, either in the acute stage of infection or following reactivation, placed into the ear pocket resulted in spread of the virus from this site to the nervous system of BALB/c scid mice, resulting in encephalitis and death. Additionally, injection of 10 viral particles into ganglion grafts resulted in viral infection and death of 50% of BALB/c scid mice, demonstrating the sensitivity of this *in vivo *system and confirming that the subcutaneous ear site is not a sequestered site that prevents the escape of infectious virus.

## Conclusion

It is concluded that measurable infectious virus is not produced under the conditions of these experiments. Thus, the technical approach used here appears to be a valid and sensitive measure of the presence of infectious virus. The results of this study reveal that molecular reactivation, *i.e.*, expression of HSV-1 genes and production of glycoproteins during latency, occurs in mice and extends this observation to establish that molecular reactivation does not necessarily lead to the production of infectious viral particles. The approach used in this investigation opens up new vistas for studying herpesvirus latency and reactivation.

## Methods

### Mice

Female BALB/cJ and BALB/c scidJ mice at 5 weeks of age (The Jackson Laboratory, Bar Harbor, ME) were used. Confirmation that the BALB/c scid mice were immune deficient was achieved by performing flow cytometric analysis of spleen cells for CD3^+ ^T cells and membrane immunoglobulin-positive cells. No evidence of the presence of T or B lymphocytes in the BALB/c scid mice sacrificed throughout the course of this study was obtained (data not shown). Animals studies were approved by the Louisiana State University Health Sciences Center Institutional Animal Care and Use Committee (IACUC). All animals were provided with food and water ad libidum and were cared for according to the NIH Guidelines on the Care and Use of Animals in Research.

### Virus

The McKrae strain of HSV-1, a strain which is known to spontaneously reactivate in rabbits, was propagated in and titered on Vero cells (American Type Culture Collection, Manassas, VA). At the time of infection, the virus stock was thawed and diluted so as to deliver 1 × 10^5 ^PFU in 4 μl of culture medium. BALB/c mice to be infected were anesthetized, their corneas were lightly scratched in a cross-hatched pattern, and 4 μl of the viral suspension was placed on the surface of each eye. In order to ensure survival, infected animals each received 0.1 ml of pooled human serum (Chemicon International, Temecula, CA) intraperitoneally at the time of infection. At 3 and 5 days after infection, the ocular surface of the animals was swabbed and tested for the presence of infectious virus by the viral plaque assay. Animals not giving evidence of infection were excluded from the study.

### Analysis of the trigeminal ganglion for infectious virus during latency

Thirty-five BALB/c strain mice were infected with the McKrae strain of HSV-1 by the topical ocular route as described above. Five days after infection, the eyes of all animals were swabbed for the determination of infectious virus. Also on day 5, five animals were killed and their trigeminal ganglia were separately homogenized in 0.5 ml of tissue culture medium. The ganglion homogenates were tested for infectious virus on Vero cell monolayers in 24-well tissue culture plates. In this experiment, no attempt was made to quantitate infectious virus, but only to note the presence or absence of infectious virus in the ganglia. Eye swabs and trigeminal ganglion homogenates were similarly tested from five additional animals killed at each of the following time points: 10, 20, 30, 50, 70, and 100 days after infection.

### Determination of the sensitivity of the assay for infectious virus in the ganglion

Groups of 10 uninfected BALB/c mice were sacrificed and their trigeminal ganglia removed intact and placed into tissue culture medium. The 20 ganglia from each group of mice were positioned under a stereoscopic microscope and each ganglion injected with a 5 μl volume of culture medium containing 100, 50, 10, 5, or 1 PFU of the McKrae strain of HSV-1. Ten ganglia from each group were immediately homogenized in 0.5 ml of culture medium, the homogenate clarified by centrifugation at 8000 × g in a microcentrifuge, and the supernatant plated on Vero cells in 24-well plates. Following viral attachment, the supernatant was removed and a 0.5% methylcellulose overlay was placed in each well. The plates were incubated for 2 days and plaques counted. The remaining 10 ganglia were transplanted into subcutaneous ear pockets in BALB/c scid mouse recipients as described below. The mice were observed daily for signs of viral pathogenesis and death.

### Ganglion transplantation

Recipient mice were anesthetized with a mixture of ketamine and xylazine and positioned under a stereoscopic dissecting microscope such that the entire ear pinna could be seen at 10× magnification. The tip of the ear was gently grasped with sterile forceps and an incision made in the dorsal skin surface with a sterile lamellar blade (Wilson Ophthalmic, Mustang, OK). The lamellar blade was gently eased below the surface of the epithelium with a side-to-side and insertion-retraction motion, creating a pocket approximately 3 mm wide and 7 mm deep. Individual ganglia were gently inserted into the ear pockets so as to allow the open end of the ear pocket to close over the graft and self-seal, thus enclosing the ganglion in the pocket and avoiding the need for sutures. One application of neomycin and polymixin sulfate ointment externally was adequate to prevent bacterial infection. The recipient animals were returned to their cages for recovery from the anesthetic and the external condition of the graft site was observed daily to ensure the success of the transplant.

Three ganglion transplantation protocols were performed:

1) Acute Protocol: BALB/c mice and BALB/c scid mice received trigeminal ganglion grafts from BALB/c donors that had been infected 5 days previously with McKrae strain HSV-1. Recipient mice were observed for evidence of viral infection, development of tissue pathology at the site of the transplant, signs of morbidity, and death. At the time of sacrifice or the time of death, serum was collected for testing for antibodies to HSV-1.

2) Latent Protocol: Trigeminal ganglia from BALB/c mice that had been infected 35 days previously with McKrae strain HSV-1 were transplanted into BALB/c and BALB/c scid mice as described above. The recipient mice were observed as described above for evidence of viral infection, viral-induced tissue pathology at the transplant site, signs of morbidity, and death. In replicates of this experiment, recipient mice were anesthetized on days 5, 14, or 28 after transplantation and the ganglion transplants removed from the skin pockets and placed in tissue culture to test for the presence of latent, reactivatable herpesvirus in the transplanted ganglion. For *in vitro *incubation, ganglia from latent BALB/c mice and explanted ganglion transplants were incubated in separate wells of 12-well culture plates containing Dulbecco's modified Eagle's medium (DMEM) supplemented with 10% fetal bovine serum (FBS) and an antibiotic/antimycotic mixture (GIBCO, Carlsbad, CA). The culture medium in each well was assayed for infectious virus on Vero cell monolayers at 1, 3, 5, 7, 10, 14, and 21 days of incubation.

3) Reactivation Protocol: Trigeminal ganglia latent with the McKrae strain of HSV-1 obtained from BALB/c mice were incubated in tissue culture for 3 days, and then transplanted into BALB/c and BALB/c scid recipients. The mice were observed for evidence of viral infection, virus-induced tissue pathology at the transplant site, signs of morbidity, and death. The recipients were tested for the development of antiviral antibody by ELISA.

### ELISA for serum antibody

Serum was collected from the BALB/c and BALB/c scid animals at the time of sacrifice. ELISA plates were coated with a cell culture lysate from Vero cells infected 18 hours previously with McKrae strain HSV-1. This lysate contains a mixture of HSV-1 antigens and has been used in previous studies [30,31]. Equal numbers of ELISA plate wells were coated with a cell culture lysate of uninfected Vero cells. The plate wells were washed three times with Tris buffered saline (TBS) and 1:50 dilutions of each serum were tested in quadruplicate for reactivity with the infected and uninfected cell lysates. Binding of serum anti-HSV antibody was detected with a secondary rabbit anti-mouse IgG antibody coupled to horseradish peroxidase (Jackson ImmunoResearch Laboratories, Inc., West Grove, PA). Following washing, the plate wells were incubated in tetramethylbenzidine substrate (DakoCytomation, Inc., Carpinteria, CA) for 15 minutes at room temperature and the reaction stopped with 1 M sulfuric acid. The optical densities were read at 450 nm in a plate reader and the optical density values for each serum sample tested on the infected cell lysate were corrected by subtraction of the optical density obtained with the serum on the uninfected cell lysate.

### Footpad swelling assay for delayed type hypersensitivity

The same infected cell lysate used to coat the ELISA plates was treated with UV light for 10 minutes to inactivate infectious virus. Mice were anesthetized with a mixture of ketamine and xylazine. The left hind footpads were injected with 10 μl of the treated, infected lysate and the right hind footpad received 10 μl of the uninfected cell lysate. At 24 hours, the footpad swelling response was measured using a spring-loaded micrometer gauge (Starett, Inc., Athol, MA). Four measurements were made of each right and left footpad. Delayed-type hypersensitivity reactions were calculated as follows: specific footpad swelling = (24 hr measurement of left footpad - 0 hr measurement of left footpad) - (24 hr measurement of right footpad - 0 hr measurement of right footpad) × 10^3 ^mm. In each experiment, in addition to the test animals, mice not immune to HSV-1 and mice immunized with UV-inactivated virus were used as negative and positive controls.

### Histology and immunohistochemical staining

Animals selected at random were sacrificed and the portion of the ear containing the ganglion transplant site was frozen and sectioned in a cryotome. The sections (10 μm) were placed on microscope slides and fixed in cold acetone for 5 minutes. Representative sections were stained with hematoxylin and eosin for histopathologic examination. Additional sections were stained for cells expressing HSV-1 antigens. A direct staining method employing a polyclonal, horseradish peroxidase-conjugated rabbit anti-HSV-1 antibody (DakoCytomation) was used. The antibody was diluted 1:200 in TBS containing 1% bovine serum albumin. Following incubation for 1 hour, the slides were washed in three washes of TBS for 5 minutes each and then incubated in the substrate consisting of diaminobenzidine and hydrogen peroxide (Pierce Biotechnology, Inc., Rockford, IL). Color development was stopped after 5 minutes and the sections were counterstained with methyl green. Control slides were incubated in an irrelevant peroxidase-labeled rabbit antibody followed by the substrate.

### Cell viability in ganglion transplants

Transplanted ganglia were removed from ear pockets at 5, 14, 28, and 65 days after transplantation. Each ganglion was teased apart with forceps in 2 ml of calcium/magnesium-free Hank's balanced salt solution (GIBCO) containing 10 U DNase, 0.1 mg/ml dispase, 0.1 mg/ml collagenase, and 0.1 mg/ml trypsin. The tissue fragments were incubated with gentle stirring for 15 minutes at 37°C. The cells and tissue clumps were gently triturated and washed two times in DMEM/10% FBS. The cells were resuspended in 2 ml DMEM/10% FBS containing 1 μg/ml of Hoechst 33342 vital DNA stain (H342, Calbiochem, La Jolla, CA) for 15 minutes at 37°C. The cells were washed twice in DMEM/FBS and resuspended in 2 ml of medium. Living and dead cells were differentiated with the addition of 0.1% trypan blue, which quenches the H342 fluorescence of dead cells [32]. The cells were placed onto clean microscope slides, coverslipped, and immediately examined on a Nikon E600 fluorescence microscope with a UV-2E/C (330–380 excitation; 435–485 barrier) filter. The cells were categorized as either small (10–50 μm in diameter, non-neurons) or larger than 50 μm in diameter (neurons). For each suspension, 10 microscopic fields at 100× magnification were examined, and the total number of cells, the numbers of small and large cells, and the numbers of fluorescing (*i.e.*, not quenched, viable) cells in each of the two size ranges were determined.

### Statistical analysis

Analysis of numerical data and statistical analyses were performed with Microsoft Excel (Redmond, WA), Modstat (Modern Microcomputers, Mechanicsville, VA), and CoStat (Cohort Software, Monterey, CA). Fisher's exact test was used to compare the differences in survival frequencies between groups of mice. *P *values less than 0.01 were considered significant.

## Competing interests

The author(s) declare that they have no competing interests.

## Authors' contributions

BMG carried out the ganglion transplantation experiments and immunoassays. WPH carried out the viral infections and plaque assays. BMG and WPH conceived of the study. BMG wrote the manuscript.
